# Salvianolic Acid B Alleviates Heart Failure by Inactivating ERK1/2/GATA4 Signaling Pathway after Pressure Overload in Mice

**DOI:** 10.1371/journal.pone.0166560

**Published:** 2016-11-28

**Authors:** Juan Yu, Renshan Chen, Yafang Tan, Jiashin Wu, Jianyong Qi, Minzhou Zhang, Weiwang Gu

**Affiliations:** 1 Laboratory Animal Center, Southern Medical University, Guangzhou city, Guangdong province, China; 2 AMI Key Laboratory of Chinese Medicine in Guangzhou, Guangdong Province Academy of Chinese Medicine, Guangdong Province Hospital of Chinese Medicine, 2^nd^ Affiliated Hospital of Guangzhou University of Chinese Medicine, Guangzhou city,Guangdong province, China; 3 Animal Laboratory, Guangdong Province Academy of Chinese Medicine, Guangzhou city, Guangdong province, China; 4 Department of Pharmaceutical Sciences, College of Pharmacy, Northeast Ohio Medical University, Rootstown, Ohio, Unitd States of America; Niigata Daigaku, JAPAN

## Abstract

**Background:**

Heart failure(HF) is a dangerous disease that affects millions of patients. Radix Salvia is widely used in Chinese clinics to treat heart diseases. Salvianolic acid B(SalB) is the major active component of Radix Salvia. This study investigated the mechanisms of action and effects of SalB on HF in an experimental mouse model of HF.

**Methods:**

We created a mouse model of HF by inducing pressure overload with transverse aortic constriction(TAC) surgery for 2 weeks and compared among 4 study groups: SHAM group (n = 10), TAC group (n = 9), TAC+MET group (metprolol, positive drug treatment, n = 9) and TAC+SalB group (SalB, 240 mg•kg^-1^•day^-1^, n = 9). Echocardiography was used to evaluate the dynamic changes in cardiac structure and function *in vivo*. Plasma brain natriuretic peptide (BNP) concentration was detected by Elisa method. In addition, H9C2 rat cardiomyocytes were cultured and Western blot were implemented to evaluate the phosphorylation of ERK1/2, AKT, and protein expression of GATA4.

**Results:**

SalB significantly inhibited the phosphorylation of Thr202/Tyr204 sites of ERK1/2, but not Ser473 site of AKT, subsequently inhibited protein expression of GATA4 and plasma BNP(P < 0.001), and then inhibited HF at 2 weeks after TAC surgery.

**Conclusions:**

Our data provide a mechanism of inactivating the ERK1/2/GATA4 signaling pathway for SalB inhibition of the TAC-induced HF.

## Introduction

Heart failure(HF) affects more than 37.7 million patients in the world **[1]**. Nearly 17.3 million people died from heart diseases in 2013, which represents a 41% increase from 1990 **[2]**. The increase in heart diseases burden is mainly due to ageing population and HF **[3]**. Clinically, patients with HF are commonly treated with pharmacological therapies to control the symptoms, such as angiotensin-converting enzyme inhibitors, angiotensin receptor blockers, beta-blockers, and mineralocorticoid receptor antagonist, etc. However, there are no effective cures for HF and the above drugs also have extensive side-effects and drug-resistance. Due to high morbidity and mortality of HF, there is a tremendous need for new and safe treatments to improve outcomes in patients with HF.

**Fig 1:**
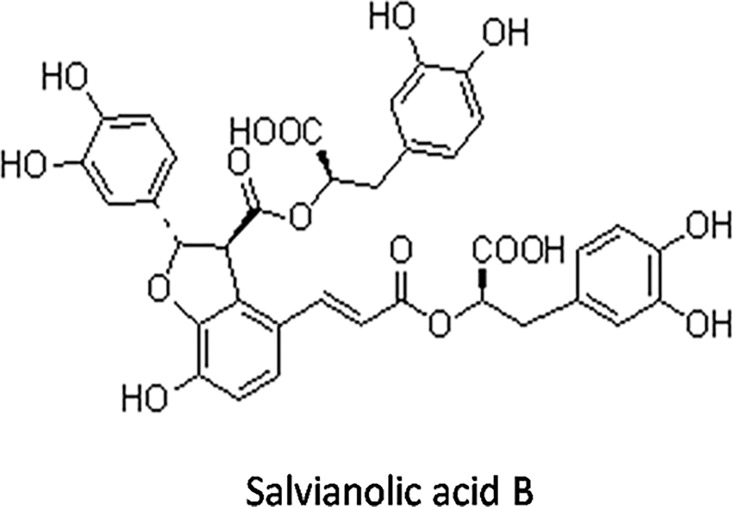
Chemical structure of Salvianolic acid B.

Salvianolic acid B (SalB), a polyphenolic acid (Fig 1), is a main water-soluble active component of Radix Salvia, which has been used widely in Traditional Chinese Medicine clinical practices. Although Radix Salvia is clinically effective in treating multiple diseases, including heart disease, pulmonary fibrosis, and malignant tumors, its most common uses are to treat cardiovascular diseases, including HF. Experiments have demonstrated that SalB could reduce ischemia-reperfusion injury, myocardial infarction, and cardiac remodeling **[4]**. Several signaling pathways are participated in SalB's actions, e.g., Akt/mTOR/4EBP1, p38 mitogen-activated protein kinase (MAPK) /ATF2, and ERK1/2 signaling pathways, via up-regulating silent mating type information regulation 2 homolog 1 and inhibiting high mobility group box-1 protein, inducing microRNA-152 and attenuating DNA methyltransferase 1-mediated Patched1 methylation, etc **[5–7]**. Although SalB is effective clinically, its role and the mechanisms of SalB's action on HF are still unclear. To clarify the underlying mechanisms, the present study investigated the effect of SalB on transverse aortic constriction(TAC)-induced HF in mice.

## Methods

### Animals and Reagents

This study was performed in accordance with the guidelines and with approval from the Institutional Animal Care and Use Committee of Guangdong Province Hospital of Chinese Medicine, Guangzhou University of Chinese Medicine, and with the Guide for the Care and Use of Laboratory Animals published by the National Academy of Sciences (8^th^ edition, Washington DC, 2011).

Ten to twelve weeks old male wild-type C57BL/6J mice (25 ± 5 g body weight) were obtained from the Experimental Animal Center of Guangdong Province. SalB was purchased from Chengdu MUST Bio-technology Corp. (Chengdu, China), batch number: MUST-15081916. Isoproterenol (ISO), metoprolol (MET), and pentobarbital sodium were purchased from Sigma Chemical (St Louis, MO, USA), fetal bovine serum (FBS) was purchased from Hyclone (Logan,UT, USA). 10% Neutral Buffered Formalin was purchased from WEX Corp. (Guangzhou, China). Brain natriuretic peptide (BNP) Enzyme-linked immunosorbent assay (ELISA) kit was purchased from Immuno-Biological laboratories Corp. (Hamburg, Germany). Other reagents were obtained from commercial suppliers.

### TAC surgery

We constructed a mouse model of HF by TAC surgery to impose pressure overload, using a proven protocol that was published previously **[8]**. Similar TAC model was also used successfully by Sadoshima et al. **[9].** In brief, increased pressure in the transverse thoracic aorta was induced by means of TAC. Male mice (C57BL/6J, 10 to 12 weeks old, 25 ± 5g body weight, from the Experimental Animal Center of Guangdong Province) were anesthetized with pentobarbital sodium (60mg/kg IP, Sigma-Aldrich Corp). Additional doses of pentobarbital were given as needed to maintain anesthesia during the protocol. The mice were orally intubated with 20-gauge tubing and ventilated (Harvard Apparatus Rodent Ventilator, model 687) at 110 breaths per minute (0.2mL tidal volume). A 3mm center thoracotomy was created. The transverse aortic arch was ligated (7–0 Prolene) between the innominate and left common carotid arteries with an overlying 28-gauge needle, and then the needle was removed, leaving a discrete region of stenosis. The chest was closed, and the pneumothorax was evacuated. The mice were removed from the ventilator, kept warm with heat lamps, given fluids (1.0–1.5 ml of 5% dextrose in water intraperitoneally), allowed 100% oxygen via nasal cone, and monitored at intervals of 30 minutes. No complication or mortality was observed after banding, and thus, all operated mice were included in the experimental groups. Some mice were subjected to a sham operation in which the aortic arch was visualized but not banded.

### Protocol

Based on literature **[10]**, we choose 240 mg/kg dosage for mice by intragastric administration (i.g) daily. Mice were assigned to four groups: SHAM, TAC (HF model), TAC + MET (metoprolol, positive drug treatment group, 100 mg/kg/d, i.g), and TAC + SalB (the aim group). Mice in SHAM group received saline i.g and all the surgery except constricting the aorta; mice in TAC group were subjected to saline i.g and TAC surgery; MET mice received metoprolol i.g and TAC surgery; SalB mice received SalB i.g and TAC surgery.

### Echocardiography

*In vivo* left ventricular (LV) function and LV structure were assessed by measuring fraction shortening (FS), and left ventricular diastolic posterior wall thickness (LVPWd), recorded by echocardiography using a Vevo 770 echocardiography system (Visual Sonics, Toronto, Canada) with a 30 MHz linear array transducer **[11]**. Briefly, Animals were anesthetized with inhaling isoflurane/oxygen, once the short-axis two-dimensional (2D) image of the left ventricle was obtained at the papillary muscle level, 2D guided M-mode images crossing the anterior and posterior walls were recorded. Parameters measured digitally on the M-mode trace were the LVPWd and inner dimension of diastolic or systolic left ventricles (LVIDd and LVIDs), and FS = (LVIDd–LVIDs)/LVIDd], LVVd = ((7.0 / (2.4 + LVIDd))×LVIDd^**3**^, LVVs = ((7.0 / (2.4 + LVIDs)) ×LVIDs^**3**^.

For aortic banding studies, to evaluate the degree of stenosis, the pressure gradient across the constriction was assessed using Doppler echocardiography **[12]**. A no imaging Doppler pencil transducer (continuous wave) was placed at the apex and orientated towards the proximal ascending aorta. The peak velocity (in meters per second) was measured, and the maximum instantaneous gradient (millimeters of Hg) was calculated using the following Bernoulli equation: Aortic Pressure Gradient(AoPg) = 4 × (velocity)^2^.

### HW assessment and histological examination

At the completion of the experiment, animals were euthanized and their hearts were removed, the left ventricle was quickly separated from the atria and right ventricular free wall and their heart [left ventricle + right ventricle] weights (HW) and body weights (BW) were determined. Then, left ventricles were fixed overnight in 4% paraformaldehyde before embedding in paraffin. Sections of 5 μm were prepared, and stained with hematoxylin-eosin (HE) for evaluation of myocyte hypertrophy.

Cardiomyocytes from LV cross sections were stained with HE, and mean values from each mouse were calculated by use of the measurements from 60 to 80 cells from an individual mouse using light microscopy at × 400 magnification. Digital photographs were obtained by using a color image analyzer (QWin Colour Binary 1, LEICA).

### Rat H9C2 Cardiomyocytes Cell Culture

Rat H9C2 cardiomyocytes cell line was obtained from the American Type Culture Collection (ATCC,Manassas,VA,USA).The H9C2 cells were maintained in DMEM supplemented with 10% fetal calf serum at 37°C in CO_2_ incubation. The medium was replaced every 2–3 days, and cells were sub-cultured or subjected to experimental procedures at 80–90% confluence.

### Western blot analysis

The protein of H9C2 cells was extracted with RIPA Lysis Buffer (Beyotime, China) containing 1% phenylmethanesulfonyl fluoride on ice. The protein concentration was detected according to bicinchoninic acid Kits. We mixed 30 μg proteins with 5× SDS-PAGE sample loading buffer. Following heating at 95°C for 5min, denatured proteins were subjected to 10% Tris-glycine gel and transferred electrophoretically to polyvinylidene difluoride membranes (Bio-Rad). Then 5% Bovine serum albumin in Tris-buffered saline with 0.1% Tween 20(TBST) was used to block non-specific sites at room temperature for 1h. The membranes were incubated with the primary antibodies overnight at 4°C and washed with TBST three times. Secondary HRP-conjugated antibodies (1:4000) were incubated with the membranes for 1 h at 37°C. Autoradiographs were quantitated by a densitometry Science Imaging system (Bio-Rad, Hercules, CA). Data were analyzed by Quantity One (Bio-Rad, USA). Following antibodies were used in this study: anti-phospho-ERK1/2 (Thr202/Tyr204) (Cell Signaling Technology, Beverly, MA,USA), anti-phospho-PKB(Ser473) (Cell Signaling Technology), anti-H3(Cell Signaling Technology), anti-ERK1/2(Santa Cruz Technology, Delaware, CA, USA), anti-GAPDH(Santa Cruz Technology), anti-eIF-5 (Santa Cruz Technology), and anti-GATA4(Selleckchem Technology, Houston,TX, USA).

### BNP ELISA Analysis

At the time of sacrifice and under anesthesia, 1 ml of blood was collected from the inferior vena cava of each mouse and immediately centrifuged at 3000 revolutions per minute for 15 minutes. The plasma supernatant was recovered and kept at −80°C until the date of BNP concentration measurement by the ELISA technique using a specific BNP kit (IBL immunobiological laboratories, Hamburg,Germany).

### Statistical analysis

Data are presented as mean ± S.E.M. Statistical analysis was performed by one-way or two-way analysis of variance followed by Turkey’s method or unpaired two-tailed Student’s t-tests. Results were considered statistically significant at *P <* 0.05.

## Results

### SalB increased cardiac systolic function after pressure overload in mice

**Fig 2:**
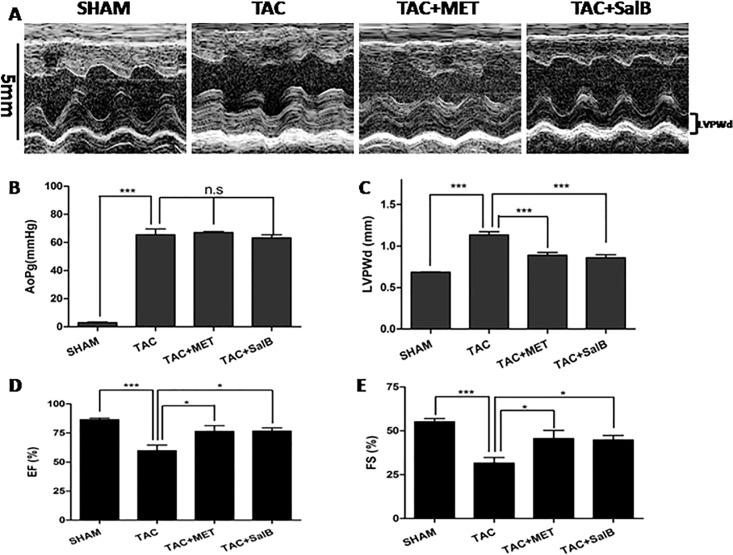
Functional Characterization of the 4 groups (SHAM, TAC, TAC+MET, and TAC+SalB mice) at 2 weeks after TAC surgery. (A) Representative echocardiograms used to measure the LV wall thickness during cardiac cycles. (B) AoPg (C) LVPWd (D) EF (E) FS measured by echocardiography were compared among the SHAM (n = 10),TAC(n = 9),TAC+MET(n = 9), and TAC+SalB (n = 9) groups. * *P <* 0.05, *** *P <* 0.001, and n.s indicates no significant differences.

We evaluated the mouse TAC-induced HF model with echo 1-mode imaging of left ventricle(LV)(a typical example is shown in Fig 2A). As shown in Table 1, there were no significances in heart rates (HR) among the four groups (*P >*0.05). AoPg were much higher in TAC, TAC+MET, and TAC+SalB groups compared with SHAM group(Fig 2B, TAC, 65.56 ± 4.07 mmHg; TAC+MET, 67.12 ± 0.68 mmHg; TAC+SalB, 63.26 ± 2.19 mmHg; SHAM, 3.029 ± 0.40 mmHg, *P <*0.001, respectively), while AoPg had no significant differences among the TAC, TAC+MET, and TAC+SalB groups (*P >*0.05). Therefore, the TAC models were successful and stable. In order to explore whether SalB has any independent effects on heart function or myocardial injury, we compared the SHAM + SalB with SHAM group using both mice and rat animals, there were no significant differences in heart function(EF,FS, the ratio of LW/TL, Lung/TL, and Liver/TL), structural changes(LVPWd, LVIDd, HW and LVW), and myocardial injury (cTnI,CK,CK-MB) between the SHAM + SalB and SHAM groups (detailed in S1 Fig and S1 Table). At the end of 2 weeks, LVPWd were increased significantly in the TAC group(Fig 2C, TAC, 1.13 ± 0.04 mm vs. SHAM, 0.69 ± 0.01 mm, *P <*0.001), while increased much less in the TAC+MET and TAC+SalB groups(TAC+MET, 0.89 ± 0.03 mm vs. TAC+SalB, 0.86 ± 0.04 mm, *P <* 0.05). These data suggested both metoprolol and SalB inhibited cardiac remodeling in ventricular wall thickness following the TAC-induced HF that the inhibitive effects were stronger by SalB than by metoprolol.

**Table 1:** Echocardiographic data of the four models.

	SHAM	TAC	MET	SalB
HR(bpm)	544.2±24.3	543.7±22.9	535.7±34.5	507.9±82.6
AoPg(mmHg)	3.029±0.40	65.56±4.07***	67.12±0.68***	63.26±2.19***
LVAWd(mm)	0.684±0.01	1.045±0.05***	0.911±0.05 ^###^	0.869±0.03 ^###^
LVAWs(mm)	1.300±0.06	1.359±0.08	1.326±0.07	1.327±0.04
LVIDd(mm)	3.226±0.08	3.486±0.10	3.205±0.17	3.339±0.14
LVIDs(mm)	1.440±0.05	2.393±0.16 ***	1.754±0.19 ^#^	1.834±0.09 ^#^
LVPWd(mm)	0.685±0.01	1.134±0.04***	0.891±0.03 ^###^	0.860±0.04 ^###^
LVPWs(mm)	1.416±0.03	1.367±0.05	1.348±0.05	1.351±0.07
EF(%)	86.57±1.35	59.89±4.79 ***	76.47±4.94 ^#^	76.74±2.71 ^#^
FS(%)	55.23±1.73	31.67±3.11 ***	45.65±4.63 ^#^	44.82±2.58 ^#^
LVM(mg)	66.98±2.60	147.4±7.96 ***	98.82±9.73 ^###^	97.79±5.37 ^###^
LVMc(mg)	53.58±2.08	117.9±6.37 ***	79.06±7.78 ^###^	78.23±4.30 ^###^
LVVd(mm^3^)	42.06±2.38	50.78±3.33	42.21±5.31	46.18±4.68
LVVs(mm^3^)	5.555±0.54	20.97±3.60 ***	10.35±2.68 ^##^	10.49±1.31 ^#^

All values were expressed as mean ± standard error of mean.

*P < 0.05

**P < 0.01

***P < 0.001 when compared with SHAM.

^**#**^*P <* 0.05

^**##**^*P <* 0.01

^**###**^*P <* 0.001 when compared with TAC.

Moreover, EF was significantly reduced in TAC mice(Fig 2D, SHAM, 86.57 ± 1.35% vs.TAC, 59.89 ± 4.79%, *P <*0.001). FS also reduced in TAC group(Fig 2E, SHAM,55.23 ± 1.73% vs. TAC, 31.67 ± 3.11%, *P <*0.001). Both of these results supported successful induction of HF in the TAC group. In contrast, both EF and FS were increased in the TAC+MET and TAC+SalB groups (*P <*0.01), suggesting that metoprolol and SalB can enhance the cardiac systolic function, and reverse cardiac remodeling from HF.

### Anatomic analysis of the HF inhibition by SalB

**Fig 3:**
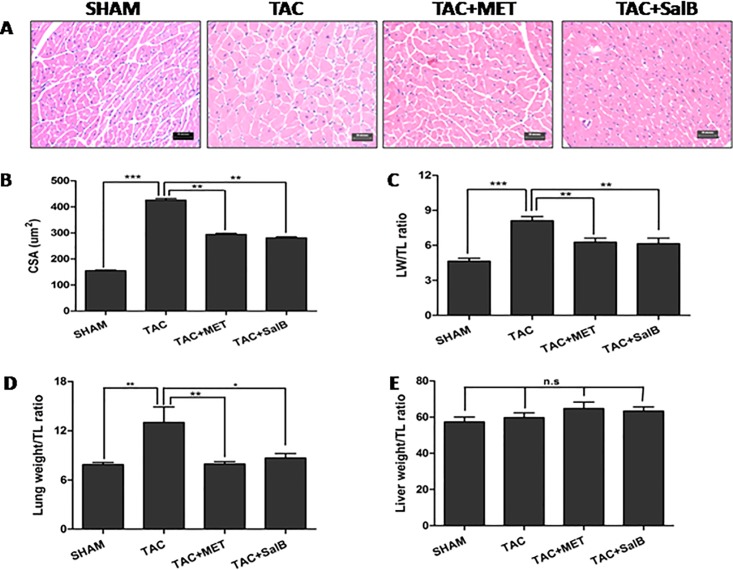
Histological sections and anatomical data from the 4 groups(SHAM, TAC, TAC+MET, and TAC+SalB mice) at 2 weeks after TAC surgery. (A) Example of the four models stained with hematoxylin-eosin (×400 magnification, Scale bar, 20 μm). (B) The LV cross section area(CSA) (C)The ratio of LV weight(LW) to tibial length(TL) (D) The ratio of lung weight to TL (E) The ratio of liver weight to TL were compared. * *P <*0.05, ** *P <*0.01, *** *P <*0.001, and n.s indicates no significant differences.

To further characterize the effect of SalB on HF, we performed microscopic anatomic analysis on HE stained thin sections of ventricle after sacrificed the mice and statistically compared the 4 groups. As shown in Fig 3A and 3B, at the end of 2 weeks, the cross-section area(CSA) of cardiomyocytes were much bigger in TAC than SHAM mice (425.5 ± 6.12 μm^2^ vs. 154.6 ± 3.0 μm^2^, *P <*0.001), but less prominent in TAC+MET and TAC+SalB mice(293.8 ± 4.49μm^2^ vs. 281.2 ± 3.62μm^2^, *P <*0.001, Fig 3B). As shown in Table 2, there were no significant differences in body weight among the 4 groups (P >0.05). Heart weights(HW) were significantly increased in the TAC mice, compared with SHAM mice(TAC, 191.5 ± 7.02 mg vs. SHAM,113.7 ± 3.94mg, *P <*0.001). The ratio of left ventricular weight(LVW) to tibial length(TL) were increased in TAC mice compared with SHAM mice(Fig 3C, TAC, 8.10 ± 0.37 vs. SHAM, 4.64 ± 0.25, *P <*0.01). Moreover, Lung weight and the ratio of lung weight to TL (Lung/TL) were found to be significantly increased in TAC mice(Fig 3D, Lung: SHAM, 137.9 ± 4.95mg vs. TAC, 226.4 ± 31.8mg, *P <*0.05; Lung/TL: SHAM, 7.870 ± 0.27 vs. TAC, 13.00±1.89, *P <*0.05, respectively), which suggested occurrence of lung edema in the TAC mice. However, there were no significant differences of the ratio of Liver weight to TL(Liver/TL) between SHAM and TAC groups(Fig 3E, *P >*0.05). Taken together, these results further demonstrated that HF occurred after TAC surgery.

**Table 2:** Anatomical data of the four models.

	SHAM	TAC	TAC+MET	TAC+SalB
Number(n)	10	9	9	9
BW(g)	23.90±0.64	22.78±0.98	26.33±0.78	24.11±0.68
HW(mg)	113.7±3.94	191.5±7.02 ***	144.5±6.17 ^###^	148.4±7.84 ^###^
LVW(mg)	81.23±4.33	142.2±6.13 ***	107.4±6.10 ^##^	107.3±7.86 ^###^
Lung(mg)	137.9±4.95	226.4±31.8 **	136.0±4.85 ^##^	150.9±9.91 ^#^
Liver(mg)	1003.±45.8	1046.±52.2	1108.±56.4	110.4±43.3
TL(mm)	17.49±0.07	17.49±0.11	17.14±0.17	17.41±0.12
HW/BW	4.753±0.09	8.595±0.63***	5.549±0.36 ^###^	6.229±0.44 ^###^
LVW/TL	4.644±0.25	8.102±0.37 ***	6.274±0.35 ^##^	4.780±0.98 ^##^
Lung/TL	7.870±0.27	13.00±1.89 **	7.944±0.28 ^##^	8.663±0.55 ^#^
Liver/TL	57.39±2.69	59.68±2.69	64.78±3.52	55.43±2.38

All values were expressed as mean ± standard error of mean.

*P < 0.05

**P < 0.01

***P < 0.001 when compared with SHAM.

^**#**^*P <* 0.05

^**##**^*P <* 0.01

^**###**^*P <* 0.001 when compared with TAC.

Subsequently, we compared HW and the ratio of LVW/TL among the TAC, TAC+MET, and TAC+SalB groups. In Fig 3C, HW and LVW/TL were significantly reduced in TAC+MET and TAC+SalB mice(TAC+MET, HW, 144.5 ± 6.17mg, LVW/TL,6.274 ± 0.35; TAC+SalB, HW,148.4 ± 7.84mg, LVW/TL,4.780 ± 0.98, respectively) compared with TAC mice(HW,191.5 ± 7.02 mg, LVW/TL,8.10 ± 0.37,*P <*0.01). All together, these data demonstrated that SalB inhibited HF that normally occur in response to pressure overload.

### Phophsrylation of ERK1/2 were inhibited in SalB group after TAC surgery

**Fig 4:**
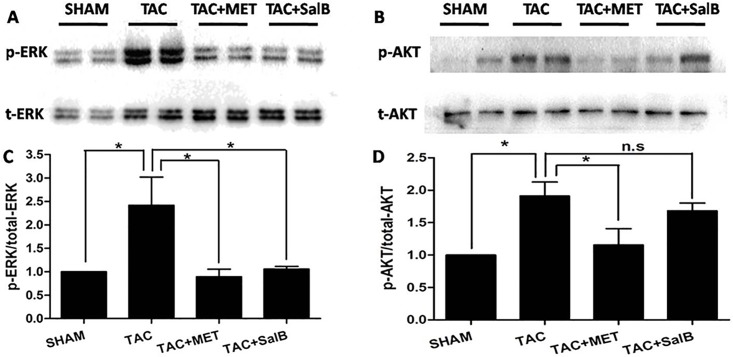
SalB inhibited the phosphorylation of ERK1/2, but not AKT *in vivo*. (A) Phosphorylated (p)-Thr202/204 extracellular signal regulated kinase (ERK) 1/2 and (B) p-Ser473 protein kinase B (AKT), and quantified data for (C) p-ERK1/2 and for (D) p-AKT. Data (mean ± SEM, n = 3) were expressed as fold changes from phosphorylated and total protein (ERK1/2, AKT). ** *P <* 0.01, *** *P <* 0.001, and n.s indicates no significant difference.

To investigate potential mechanisms of SalB's protection against the pressure-overload-induced HF, we focused on ERK1/2 and AKT, which are two major signaling pathways involved in HF **[13].** Using Western blot analysis, we found that the ERK1/2 phophsrylations of threonines at 202^th^ and tyrosine at 204^th^ sites were enhanced after TAC surgery, compared with control(Fig 4A and 4C, *P<*0.001). Moreover, the phosphorylations of threonines at 202th and tyrosine at 204th sites were reduced after metoprolol incubation, consistent with published data **[14]**. Interestingly, the phosphorylations of ERK1/2 was blocked after SalB stimulation(P<0.001, Fig 4A and 4C). Thus, these data revealed that SalB inhibited ERK1/2 phosphorylations of threonines at 202^th^ and tyrosine at 204^th^ sites. In addition, we detected the AKT phosphorylation of tyrosine at 473^th^ site, which was increased in TAC group (Fig 4B and 4D, *P<*0.01, vs. Control), but did not decrease after further SalB stimulation. Together, these data suggested that SalB reversed the ERK1/2 phosphorylations of threonines at 202^th^ and tyrosine at 204^th^ sites, but not the AKT phosphorylation of tyrosine at 473^th^ site *in vivo*.

### The Expression of GATA4 and BNP were reduced after SalB stimulation in HF mice

**Fig 5:**
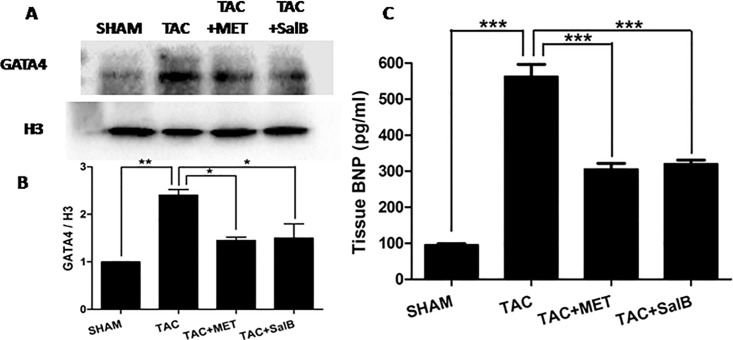
SalB inhibited the protein expression of GATA4 and BNP *in vivo*. (A) Western blot bands of the protein expression of GATA4 and H3, (B) their fold changes, and (C) the plasma contents of BNP were compared from SHAM, TAC, TAC+MET, and TAC+SalB groups. Data are mean ± SEM(*n* = 3). * *P <*0.05, ** *P <*0.01, *** *P <*0.001.

To further investigate the involvement of ERK1/2 pathway in SalB's inhibitory effects on HF, we analyzed a typical down-stream target, GATA4 zinc finger, which, in association with multiple signaling pathways, such as ERK1/2, p38, and Akt **[15, 16]**. As shown in Fig 5A and 5B, we isolated nuclear GATA4 protein from cytoplasm proteins. The expression of GATA4 in nuclear was significantly decreased in TAC+SalB group, compared with TAC only (P < 0.05). Furthermore, the serum content of BNP was increased in the mice of HF group(TAC group), and reduced significantly after additional SalB stimulation *in vivo* (*P<*0.05,Fig 5C). Thus, SalB inhibited the nuclear protein expression of GATA4 and serum BNP in response to pressure overload.

### Phophsrylation of ERK1/2 were inhibited by SalB followed ISO stimulation in H9C2 cardiomyocytes

**Fig 6:**
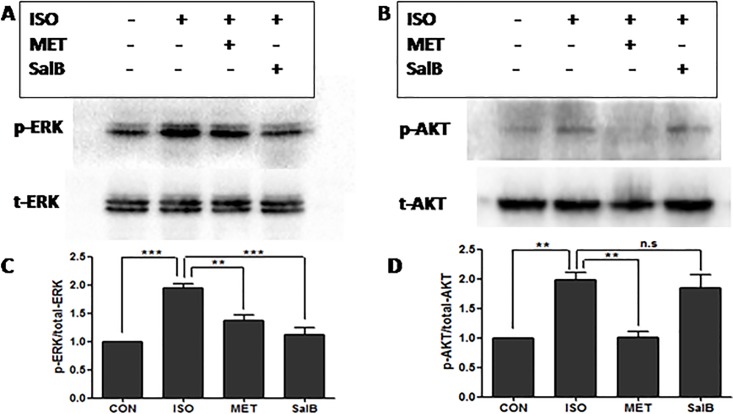
SalB inhibited the phosphorylation of ERK1/2, but not AKT *in vitro*. (A) Phosphorylated (p)-Thr202/204 extracellular signal regulatedkinase (ERK) 1/2 and (B) p-Ser473 protein kinase B (AKT), and quantified data for (C) p-ERK1/2 and for (D) p-AKT. Data (mean± SEM, n = 3) were expressed as fold changes from phosphorylated and total protein (ERK1/2, AKT).**P<0.01, *** *P<*0.001. n.s. indicates no significant difference.

To simulate the pressure-overload stress *in vitro*, we cultured rat H9C2 cardiomyocyte line with pre-incubation of 1 μM ISO for 10 minutes. Western blot experiments showed similar results in the *in vitro* cardiomyocytes as in the *in vivo* study. The phosphorylations of ERK1/2 was increased by ISO stimulation but blocked after SalB stimulation(P<0.001, Fig 6A and 6B). Also, AKT phosphorylation of tyrosine at 473^th^ site did not decrease after SalB stimulation. Together, these data further confirmed the observation that SalB reversed the ERK1/2 phosphorylations of threonines at 202^th^ and tyrosine at 204^th^ sites, but not the AKT phosphorylation of tyrosine at 473^th^ site *in vivo* and *in vitro*.

### The Expressions of GATA4 and BNP were reduced by SalB after ISO stimulation *in vitro*

**Fig 7:**
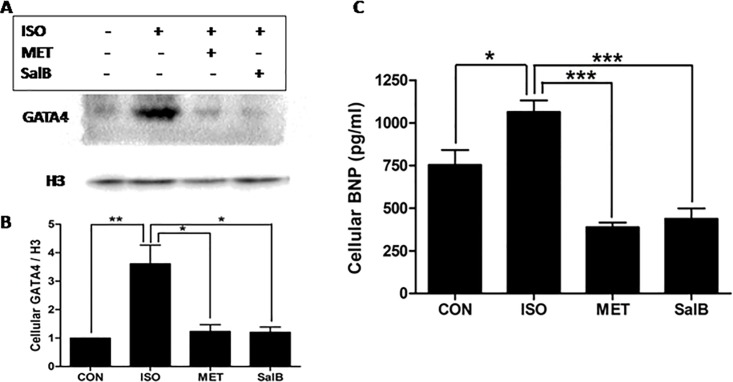
SalB inhibited protein expressions of GATA4 and BNP in vitro. (A) Western blot bands of GATA4 and H3, (B) their fold changes, and (C) the contents of BNP were compared from CON, ISO, MET, and SalB groups. Data are mean ± SEM(n = 3). * *P* <0.05, ** *P* <0.01, *** *P* <0.001.

We analyzed the expressions of GATA4 and BNP in H9C2 rat cardiomyocytes *in vitro* to further investigate the ERK1/2 pathway in SalB’s inhibitory effects on HF. As shown in Fig 7A and 7C, nuclear expression of GATA4 was significantly lower in the SalB group than the ISO stimulation-only group(P< 0.05). Therefore, SalB inhibited the ISO-stimulated expression of nuclear GATA4 protein. Moreover, BNP was increased after ISO stimulation but was reduced significantly after additional SalB stimulation *in vitro* (*P<*0.05,Fig 7B)

**Fig 8:**
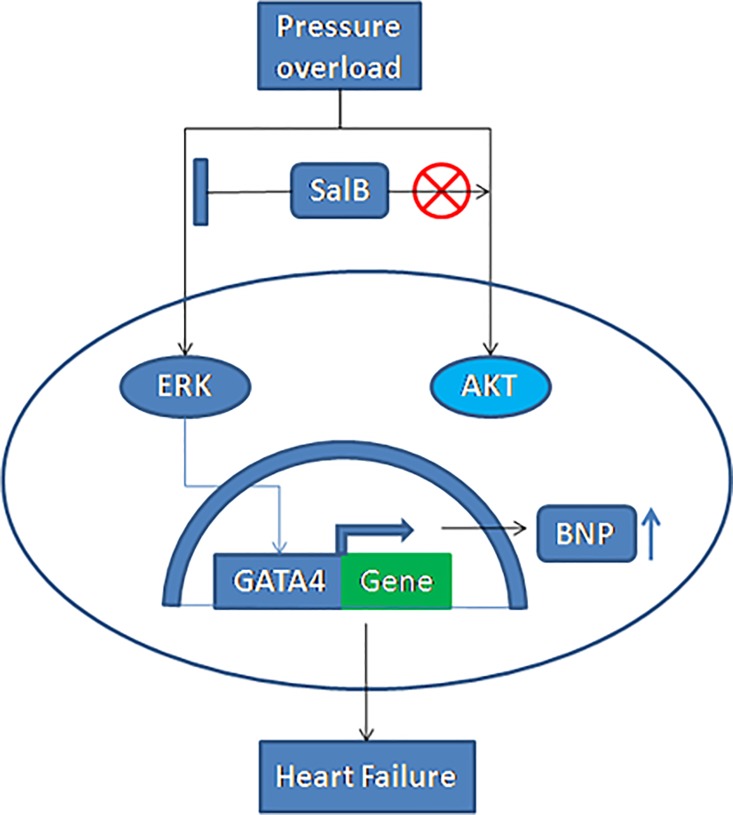
A model of the cardioprotective effects of SalB against the induction of HF by pressure stress overload. TAC (stress overload) could activate phosphorylation of the protein kinases of ERK1/2 and AKT, enhance the expression of GATA4, promote the transcription of BNP gene, and result in HF. SalB could inhibit the phosphorylation of ERK1/2,decreasing the expression of GATA4 and BNP, leading to inhibit HF. ⫿ denotes inhibition of protein kinase by SalB treatment, ⨂ denotes AKT was not participated in SalB treatment.

Based on the above observations, we formulated following working model(Fig 8):pressure overload by TAC activate MAPK1/2 and AKT, leading to HF; SalB could inhibit the phosphorylation of ERK1/2,but not PI3K/AKT pathway, decreasing the expression of transcription factor GATA4 and BNP protein translation, so as to inhibit HF.

## Discussion

The present study revealed that SalB elicited cardio-protective effects(increasing EF,FS and reverses cardiac remodeling), and thus, alleviated HF in response to pressure overload *in vivo*. These protective effects of SalB were mediated via the MAPK1/2/GATA4 signaling pathway without involvement of AKT. To the best of our knowledge, this is the first study investigating the mechanisms of SalB's protective effects against HF in response to pressure overload in mice.

### Mechanisms of ERK1/2/GATA4 in HF

HF is generally considered as a consequence of the maladaptive alterations under persistent stresses and pathological conditions, and mediated via multiple signaling pathways, such as MAPKs, PI3-K/AKT, PKCalpha, Ca2+/calmodulin-dependent kinase II, Phosphodiesterase 5, HDAC, etc **[17]**. Depletion of ERK1/2 activity significantly decreased the severity of HF in TAC mice **[18]**. Over-expression of ERK1/2 promoted the compensated cardiac hypertrophy, although did not induce HF**[19]**. PI3K/AKT signaling pathway is usually implicated in physiological cardiac hypertrophy. However, mice with targeted disruptions of AKT demonstrated a significant reduction in TAC-induced cardiac hypertrophy, which indicates that AKT is required for TAC-regulated hypertrophic responses.

GATA4 plays a key role in regulating cardiac hypertrophy and HF by binding to the promoters of BNP, ANF, alpha-MHC, and beta-MHC genes, and thus, controls their expressions in the heart **[20]**. ERK1/2 and AKT are up-streams to increase GATA4 DNA binding during acute wall stretching **[21]**. Cardiac specific GATA4 knockout(loss of function) are more resistant to development of cardiac hypertrophy and HF after agonist stimulation or pressure overload **[22]**. Gain of function by infecting mice with GATA4 recombinant adenovirus induced cardiomyocytes hypertrophy **[23].** Here we demonstrated that the expression of GATA4 in response to ISO stimulation was reduced after SalB treatment, which reveals gene regulation mechanisms of SalB on HF.

### Role of SalB in heart disease

In addition to the MAPK1/2/GATA4 signaling pathway as demonstrated in the current study, additional signaling pathways and mechanisms can also participate in the actions of SalB. SalB can increase superoxide dismutase (SOD) activity **[24]**, reduce apoptosis by PI3K/Akt and ERK1/2 signal path **[25,26]**, and thus, reduce myocardial ischemia and reperfusion injury**[27]** and brain tissue injury **[28]**. Moreover, SalB inhibits tumor necrosis factor-alpha (TNF-alpha)-induced MMP-2 upregulation in human aortic smooth muscle cells via suppression of NAD(P)H oxidase-derived reactive oxygen species **[29]**. SalB also reduces the expressions of inflammatory factors, such as interleukins (IL-1β, IL-6, and IL-8), alleviates apoptosis of vascular cells to improve heart function, reduces liver and renal fibrosis via down-regulating transforming growth factor β1 (TGF-β1) and depressing MMP-2 activity **[30]**. SalB suppresses expression of COX-2, consequently retarding growth of cancer cells as demonstrated in both *in vivo* and *in vitro* studies **[31]**. Lastly, SalB promotes stem cells proliferation, increasing the number of G2/S stage cell to form cell globe **[32]**, which can further differentiate towards nerve cells, lipid cells, or endothelial cells. In our study, we found ERK1/2 pathway participated in SalB inhibition of HF with similar mechanisms as the protective effects of SalB on myocardial ischemia and reperfusion injury.

### Significance and clinical relevance

HF is one of the major causes of human death. Current drug therapies for HF, such as angiotensin-converting enzyme (ACE) inhibitors, angiotensin receptor blockers (ARBs), beta-blockers, and mineralocorticoid receptor antagonist can only slow the progression, and have extensive side effects, including cough, vomiting, skin itching, and aggravating kidney damage. SalB has a good potential to be developed into a low side effects but effective therapy for HF. In the present study, SalB administered intragastric to mice for 2 weeks at dose up to 240 mg/kg did not induce any changes in body weight, food consumption, organ ratio, hematological change, or gross pathology of liver and kidney.

### Study limitation

It is generally considered that the development of HF takes 4 weeks or more in patients. Stress of aortic pressure gradient increases after overload, leading to compensatory cardiac hypertrophy that initially improves metabolic profile, oxygen uptake and ketogenesis, but eventually results in HF. In the present study, mice in 2 weeks after TAC surgery had typical signs of HF with significantly reduced cardiac systolic function (EF,FS) and AoPg of > 60 mmHg. Further *in vivo* animal, *in vitro*, and clinical patient studies are needed before the results of current study can be utilized in clinic treatment of patients with chronic HF.

## Conclusion

In conclusion, the current study demonstrated that SalB inhibited TAC-induced HF via an ERK1/2/GATA4 signaling pathway. The present results enhanced our understanding of the role of SalB on HF. The results suggest that SalB has the potential to be a therapeutic target, and elucidate mechanisms of the well-documented and reported effectiveness of Radix Salvia treating patients with HF.

## Supporting Information

S1 Fig Anatomic and echocardiographic data between SHAM+SalB and SHAM mice. (DOCX)

S1 Table Anatomical data and serum markers of myocardial injury between SHAM+SalB and SHAM rats. (DOCX)

## Abbreviations

TAC: transverse aortic constriction
HR: heart rate
HW: heart weight
BW: body weight
AoPg: aortic pressure gradient
LV: left ventricle
LVW: LV weight
LVAWd: LV end-diastolic anterior wall thickness
LVAWs: LV end-systolic anterior wall thickness
LVIDd: inner dimension of diastolic LV
LVIDs: inner dimension of systolic LV
LVPWd: LV end-diastolic posterior wall thickness
LVPWs: LV end-systolic posterior wall thickness
EF: eject fraction
FS: fractional shortening
LVM: mass of LV
LVMc: corrected mass of LV
LVVd: LV end-diastolic volume
LVVs: LV end-systolic volume
TL: tibial length

## References

[1] Bui AL, Horwich TB, Fonarow GC. Epidemiology and risk profile of heart failure. Nat Rev Cardiol 2011; 8: 30–41. 10.1038/nrcardio.2010.165 21060326 PMC3033496

[2] GBD 2013 Mortality and Causes of Death Collaborators. Global, regional, and national age‑sex specific all‑cause and cause‑specific mortality for 240 causes of death, 1990–2013: a systematic analysis for the Global Burden of Disease Study. Lancet. 2015;385:117–71. 10.1016/S0140-6736(14)61682-2 25530442 PMC4340604

[3] Mozaffarian D, Benjamin EJ, Go AS, Arnett DK, Blaha MJ, Cushman M, et al Heart Disease and Stroke Statistics-2016 Update: A Report From the American Heart Association. Circulation. 2016;133:e38–e360. 10.1161/CIR.0000000000000350 26673558

[4] Guo HD, Cui GH, Tian JX, Lu PP, Zhu QC, Lv R, et al Transplantation of salvianolic acid B pretreated mesenchymal stem cells improves cardiac function in rats with myocardial infarction through angiogenesis and paracrine mechanisms. Int J Cardiol. 2014;177:538–42. 10.1016/j.ijcard.2014.08.104 25189503

[5] Tang Y, Jacobi A, Vater C, Zou X, Stiehler M. Salvianolic acid B protects human endothelial progenitor cells against oxidative stress-mediated dysfunction by modulating Akt/mTOR/4EBP1, p38 MAPK/ATF2, and ERK1/2 signaling pathways. Biochem Pharmacol. 2014;90:34–49. 10.1016/j.bcp.2014.04.008 24780446

[6] Zeng W, Shan W, Gao L, Gao D, Hu Y, Wang G, et al Inhibition of HMGB1 release via salvianolic acid B-mediated SIRT1 up-regulation protects rats against non-alcoholic fatty liver disease. Sci Rep. 2015;5:16013 10.1038/srep16013 26525891 PMC4630617

[7] Yu F, Lu Z, Chen B, Wu X, Dong P, Zheng J. Salvianolic acid B-induced microRNA-152 inhibits liver fibrosis by attenuating DNMT1-mediated Patched1 methylation. J Cell Mol Med. 2015;19:2617–32. 10.1111/jcmm.12655 26257392 PMC4627567

[8] Qi J, Liu Q, Gong K, Yu J, Wang L, Guo L, et al Apocynum Tablet Protects against Cardiac Hypertrophy via Inhibiting AKT and ERK1/2 Phosphorylation after Pressure Overload. Evid Based Complement Alternat Med. 2014;2014:769515 10.1155/2014/769515 25093027 PMC4100359

[9] Shirakabe A, Zhai P, Ikeda Y, Saito T, Maejima Y, Hsu CP, et al Drp1-Dependent Mitochondrial Autophagy Plays a Protective Role Against Pressure Overload-Induced Mitochondrial Dysfunction and HF. Circulation. 2016;133:1249–63. 10.1161/CIRCULATIONAHA.115.020502 26915633 PMC4811679

[10] Wu YT, Chen YF, Hsieh YJ, Jaw I, Shiao MS, Tsai TH. Bioavailability of salvianolic acid B in conscious and freely moving rats. Int J Pharm. 2006 12 1;326(1–2):25–31. 10.1016/j.ijpharm.2006.07.003 16905282

[11] Sapra G, Tham YK, Cemerlang N, Matsumoto A, Kiriazis H, Bernardo BC, et al The small-molecule BGP-15 protects against heart failure and atrial fibrillation in mice. Nat Commun. 2014; 5:5705 10.1038/ncomms6705 25489988

[12] McMullen JR, Sherwood MC, Tarnavski O, Zhang L, Dorfman AL, Shioi T, et al Inhibition of mTOR signaling with rapamycin regresses established cardiac hypertrophy induced by pressure overload. Circulation. 2004;109:3050–5. 10.1161/01.CIR.0000130641.08705.45 15184287

[13] Bénard L, Oh JG, Cacheux M, Lee A, Nonnenmacher M, Matasic DS, et al Cardiac Stim1 Silencing Impairs Adaptive Hypertrophy and Promotes Heart Failure Through Inactivation of mTORC2/Akt Signaling. Circulation. 2016;133:1458–71. 10.1161/CIRCULATIONAHA.115.020678 26936863 PMC4829441

[14] Kovacs K, Hanto K, Bognar Z, Tapodi A, Bognar E, Kiss GN, et al Prevalent role of Akt and ERK activation in cardioprotective effect of Ca(2+) channel- and beta-adrenergic receptor blockers. Mol Cell Biochem. 2009 1;321(1–2):155–64. 10.1007/s11010-008-9929-8 18975057

[15] Li Q, Shen P, Zeng S, Liu P. TIEG1 Inhibits Angiotensin II-induced Cardiomyocyte Hypertrophy by Inhibiting Transcription Factor GATA4. J Cardiovasc Pharmacol. 2015;66:196–203. 10.1097/FJC.0000000000000265 26252173

[16] Xu X, Zhang L, Liang J. Rosuvastatin prevents pressure overload‑induced myocardial hypertrophy via inactivation of the Akt, ERK1/2 and GATA4 signaling pathways in rats. Mol Med Rep. 2013;8:385–92. 10.3892/mmr.2013.1548 23799547

[17] Burchfield JS, Xie M, Hill JA. Pathological ventricular remodeling: mechanisms: part 1 of 2. Circulation. 2013;128:388–400. 10.1161/CIRCULATIONAHA.113.001878 23877061 PMC3801217

[18] Ulm S, Liu W, Zi M, Tsui H, Chowdhury SK, Endo S, et al Targeted deletion of ERK2 in cardiomyocytes attenuates hypertrophic response but provokes pathological stress induced cardiac dysfunction. J Mol Cell Cardiol. 2014;72:104–16. 10.1016/j.yjmcc.2014.03.002 24631771 PMC4046245

[19] Bueno OF, De Windt LJ, Tymitz KM, Witt SA, Kimball TR, Klevitsky R, et al The MEK1-ERK1/2 signaling pathway promotes compensated cardiac hypertrophy in transgenic mice. EMBO J. 2000;19:6341–50. 10.1093/emboj/19.23.6341 11101507 PMC305855

[20] Molkentin JD. The zinc finger-containing transcription factors GATA-4, -5, and -6: ubiquitously expressed regulators of tissue-specific gene expression. J Biol Chem. 2000;275:38949–52. 10.1074/jbc.R000029200 11042222

[21] Tenhunen O, Sármán B, Kerkelä R, Szokodi I, Papp L, Tóth M, et al Mitogen-activated protein kinases p38 and ERK 1/2 mediate the wall stress-induced activation of GATA-4 binding in adult heart. J Biol Chem. 2004;279:24852–60. 10.1074/jbc.M314317200 15051723

[22] Oka T, Maillet M, Watt AJ, Schwartz RJ, Aronow BJ, Duncan SA, et al Cardiac-specific deletion of Gata4 reveals its requirement for hypertrophy, compensation, and myocyte viability. Circ Res. 2006;98:837–45. 10.1161/01.RES.0000215985.18538.c4 16514068

[23] Liang Q, De Windt LJ, Witt SA, Kimball TR, Markham BE, Molkentin JD. The transcription factors GATA4 and GATA6 regulate cardiomyocyte hypertrophy *in vitro* and *in vivo*. J Biol Chem. 2001;276:30245–53. 10.1074/jbc.M102174200 11356841

[24] Huang M, Wang P, Xu S, Xu W, Xu W, Chu K, Lu J. Biological activities of salvianolic acid B from Salvia miltiorrhiza on type 2 diabetes induced by high-fat diet and streptozotocin. Pharm Biol. 2015 7;53(7):1058–65. 10.3109/13880209.2014.959611 25612777

[25] Tang Y, Jacobi A, Vater C, Zou X, Stiehler M.Salvianolic acid B protects human endothelial progenitor cells against oxidative stress-mediated dysfunction by modulating Akt/mTOR/4EBP1, p38 MAPK/ATF2, and ERK1/2 signaling pathways. Biochem Pharmacol. 2014 7 1;90(1):34–49. 10.1016/j.bcp.2014.04.008 24780446

[26] Liu CL, Xie LX, Li M, Durairajan SSK, Goto S, Huang JD. salvianolic acid B inhibits hydrogen peroxide-induced endothelial cell apoptosis through regulating PI3K/Akt signaling. PLoS ONE 2007;2:e1321 10.1371/journal.pone.0001321 18091994 PMC2117346

[27] Deng Y, Yang M, Xu F, Zhang Q, Zhao Q, Yu H, et al Combined Salvianolic Acid B and Ginsenoside Rg1 Exerts Cardioprotection against Ischemia/Reperfusion Injury in Rats. PLoS One. 2015 8 17;10(8):e0135435 10.1371/journal.pone.0135435 26280455 PMC4539231

[28] Lv H, Wang L, Shen J, Hao S, Ming A, Wang X, et al Salvianolic acid B attenuates apoptosis and inflammation via SIRT1 activation in experimental stroke rats. Brain Res Bull. 2015 6;115:30–6. 10.1016/j.brainresbull.2015.05.002 25981395

[29] Zhang HS, Wang SQ. Salvianolic acid B from Salvia miltiorrhiza inhibits tumor necrosis factor-alpha (TNF-alpha)-induced MMP-2 upregulation in human aortic smooth muscle cells via suppression of NAD(P)H oxidase-derived reactive oxygen species. J Mol Cell Cardiol. 2006 7;41(1):138–48. 10.1016/j.yjmcc.2006.03.007 16713603

[30] Liu M, Zheng M, Xu H, Liu L, Li Y, Xiao W, Li J, Ma E.Anti-pulmonary fibrotic activity of salvianolic acid B was screened by a novel method based on the cyto-biophysical properties.Biochem Biophys Res Commun. 2015 12 4–11;468(1–2):214–20. 10.1016/j.bbrc.2015.10.127 26523510

[31] Pan RH, Rui GH, Yao G, Wu JN, Wu XC, Yang C. Effect of salvianolic acid B on epithelial-mesenchymal transition in rats with unilateral ureteral obstruction. Chin J Integr Tradit West Nephrol (Chin) 2008;9:779–981.

[32] Zhao Y, Guo YH, Gu XB. Salvianolic acid B, a potential chemopreventive agent for head and neck Squamous cell cancer. J Oncol 2011;2011:534548–534556. 10.1155/2011/534548 21209716 PMC3010684

