# Supplementary material for: Salvianolic Acid B Alleviates Heart Failure by Inactivating ERK1/2/GATA4 Signaling Pathway after Pressure Overload in Mice
Source: PLoS One. 2016 Nov 28;11(11):e0166560. doi: 10.1371/journal.pone.0166560 (PMC5125602; doi:10.1371/journal.pone.0166560)
Supplement: S1 Fig — (DOCX) [file pone.0166560.s001.docx]

S1 Fig. Anatomic and echocardiographic data between SHAM+SalB and SHAM mice.


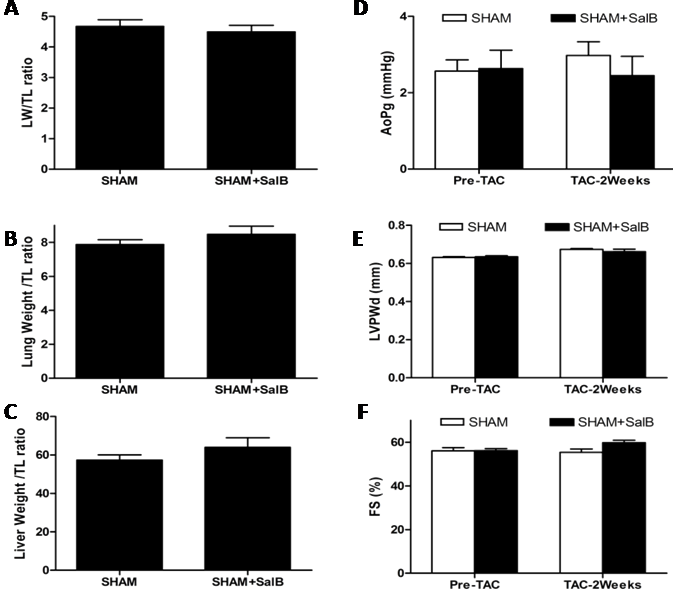


S1 Fig. SHAM + SalB and SHAM groups were compared from anatomic and echocardiaographic data in mice. There were no any significant differences in LW/TL(A),Lung/TL(B),Liver/TL(C),AoPg(D),LVPWd(E),and FS(F).
