# Supplementary material for: Salvianolic Acid B Alleviates Heart Failure by Inactivating ERK1/2/GATA4 Signaling Pathway after Pressure Overload in Mice
Source: PLoS One. 2016 Nov 28;11(11):e0166560. doi: 10.1371/journal.pone.0166560 (PMC5125602; doi:10.1371/journal.pone.0166560)
Supplement: S1 Table — (DOCX) [file pone.0166560.s002.docx]

**S2 Table. Anatomical data and serum markers of myocardial injury between SHAM+SalB and SHAM rats.**

|  |  | **SHAM** |  | **SHAM + SalB** |
| --- | --- | --- | --- | --- |
|  |  |  |  |  |
| **BW(g)** |  | **213.4 ± 4.08** | | **225.3 ± 5.48** |
| **Heart(mg)** |  | **70.50 ± 1.98** |  | **70.46 ± 2.73** |
| **Lung(mg)** | | **96.91 ± 2.37** |  | **90.56 ± 7.26** |
| **Liver(mg)** |  | **794.6 ± 30.5** |  | **967.8 ± 36.1** |
| **TL(mm)** |  | **35.42 ± 0.30** |  | **36.45 ± 0.28** |
| **HW/TL** |  | **1.991 ± 0.06** |  | **1.934 ± 0.08** |
| **Lunt/TL** |  | **2.738 ± 0.07** |  | **2.481 ± 0.19** |
| **Liver/TL** |  | **22.44 ± 0.87** |  | **26.55 ± 0.95** |
| **cTnI(pg/ml)** |  | **0.018 ± 0.01** |  | **0.009 ± 0.004** |
| **CK(pg/ml)** |  | **470.3 ± 57.1** |  | **519.7 ± 180.6** |
| **CK-MB(pg/ml)** |  | **605.7 ± 61.6** |  | **536.6 ± 63.4** |
| **AST(pg/ml)** |  | **81.00 ± 8.71** |  | **97.57 ± 6.51** |
| **LDH(pg/ml)** |  | **311.0 ± 37.6** |  | **287.0 ± 36.3** |
| **a-HBD(pg/ml)** | | **71.00 ± 9.89** |  | **94.14 ± 18.1** |
|  |  |  |  |  |

S2 Table. SHAM + SalB and SHAM groups were sacrificed after isoproterenol (85mg/kg/d) were intraperitoneal injection 2days. Heart, lung, liver, tibial length(TL) and serum markers of myocardial injury ( cTnI, CK,CK-MB, AST,LDH,a-HBD) were measured in rats. There were no any significant differences between the 2 groups.
